# Articular Cartilage Reconstruction with Hyaluronate-Based Scaffold Significantly Decreases Pain and Improves Patient’s Functioning

**DOI:** 10.3390/jcm12237342

**Published:** 2023-11-27

**Authors:** Jarosław Gryglewicz, Monika Chaszczewska-Markowska, Mateusz Dorochowicz, Jerzy Drożdż, Szymon Łukasz Dragan

**Affiliations:** Department of Orthopaedics, Traumatology and Hand Surgery, Faculty of Medicine, Wroclaw Medical University, Borowska 213, 50-556 Wrocław, Poland

**Keywords:** cartilage, lesion, osteochondral, scaffold, hyalofast, vitamin D

## Abstract

Articular cartilage lesions negatively affect patients’ well-being, causing severe pain and significantly limiting functioning. The purpose of this study was to evaluate the effectiveness of a one-stage reconstruction, performed arthroscopically using a hyaluronate-based scaffold. Pain reduction and functional improvement were assessed. The study also evaluated if postoperative vitamin D supplementation and rehabilitation protocol impact obtained outcomes. A group of 29 patients was included in a retrospective study. All the participants underwent arthroscopic reconstruction of osteochondral lesions using hyaluronate-based scaffolds. The study group used standard questionnaires to self-assess their condition before surgery and at the time of completion. Despite the aforementioned, all the participants fulfilled two original questionnaires on postoperative rehabilitation and vitamin D supplementation. Significant pain reduction (mean NRS 1.83 vs. 7.21, *p* < 0.0001) and functional improvement (mean Lysholm score 82.38 vs. 40.38, *p* < 0.0001; mean OKS 40.2 vs. 23.1, *p* < 0.0001) were found. No differences in pain reduction and functional improvement were seen between genders. The impact of post-operative rehabilitation and vitamin D supplementation on clinical outcomes was found to be statistically nonsignificant. The results obtained in this study clearly confirm the effectiveness of osteochondral reconstruction using hyaluronate-based scaffolds. The outcomes were equally favorable, regardless of postoperative rehabilitation and vitamin D supplementation.

## 1. Introduction

Articular cartilage lesions are a complex problem which clinicians are increasingly faced with. The lesions remain asymptomatic for a long time, and in a significant number of cases are found as concomitant pathologies during the diagnostic process after a joint trauma.

Hyaline cartilage is a tissue that allows joints to glide painlessly. It is composed of specialized cells (chondrocytes) and an extracellular matrix (ECM), which, aside from water (in normal cartilage, about 90% of ECM consist of water), is largely formed by collagens, the most important and common of which is type II. Fluctuations in the composition of articular cartilage can be pathological (chondromalacia), but they can also occur as a result of joint dysfunction (e.g., chondromalacia of the articular surface of the patella as a result of patellar lateral pressure syndrome) [1].

Chondral and osteochondral lesions are an important clinical problem, as they impair joint function, cause pain, and significantly accelerate osteoarthritis. Due to the lack of innervation of the articular cartilage (which enables its function), lesions of intermediate thickness often remain asymptomatic for a long time, while only irritation of the richly innervated subchondral tissue (lesions of grade III and IV according to the ICRS and Outerbridge classification) cause symptoms. Lack of vascularization determines almost no possibility of spontaneous healing of cartilage damage. The quality of articular cartilage can be regarded as an indicator of joint well-being, while any damage to it will be a factor significantly accelerating degenerative changes [2].

Therapeutic methods for suffering from joint surface damage can be divided into conservative (physiotherapy, supplementation of glycosaminoglycans, injections of hyaluronic acid and platelet-rich plasma, administration of analgesic drugs, etc.) and a variety of surgical procedures [3]. The goal of surgical treatment is to restore articular cartilage, but unfortunately, developing a method that is reproducible and enables the restoration of a physiological tissue remains unsuccessful. The scar tissue produced by the procedures only partially corresponds histologically and biomechanically to the hyaline cartilage. The expected short-term outcomes of any treatment are pain reduction and functional improvement, whilst in the long-term, the goal is to decelerate the development of osteoarthritis [4].

Several methods designed to reconstruct cartilage have been developed, which vary in surgical technique—some of them can be carried out using a minimally invasive arthroscopic method. It is important in the patient’s recovery process whether the procedure can be performed in a single operation or has to be divided into several stages (the ACI technique as well as its derivatives are two-stage procedures, the surgeries are performed several weeks/months apart). The aim of this study was to evaluate the outcome of a one-stage reconstruction procedure that combines the microfracture technique with the use of a hyaluronate-based scaffold (HYALOFAST^®^, Anika Therapeutics, Inc., Bedford, MS, USA). Of note, this is a method that does not require tissue harvesting from the initially uninjured site (as is done during mosaicplasty). The purpose of the microfractures is to stimulate the subchondral layer to deliver mesenchymal cells of the bone marrow to the area being treated, while the role of the scaffold is to secure the produced superclot in place and provide favorable conditions for formation of the regenerate [5].

In recent years, there have been studies investigating the effect of vitamin D levels on joint pain, as well as its impact on the recovery process and the therapeutic effects achieved. The available literature indicates the existence of the above-mentioned relationships, hence, we also evaluated the significance of the postoperative rehabilitation and postoperative vitamin D supplementation on obtained therapeutic effects [6].

## 2. Results

All of the 29 patients included into this study were interviewed by a clinician and asked to evaluate their current general health and functional status. The interview was conducted either during a face-to-face or tele-consultation. Each patient was asked to fulfill a questionnaire composed of pain assessment (NRS) and a complex functional evaluation (OKS and Lysholm Knee Scoring Scale). The mean evaluation time was 23.9 months after surgery (min-max 18.00–27.00 months, SD 1.84). Participants were also asked about voluntary vitamin D supplementation (this was not included in the post-operative recommendations) and the course of postoperative rehabilitation.

**Figure 1:**
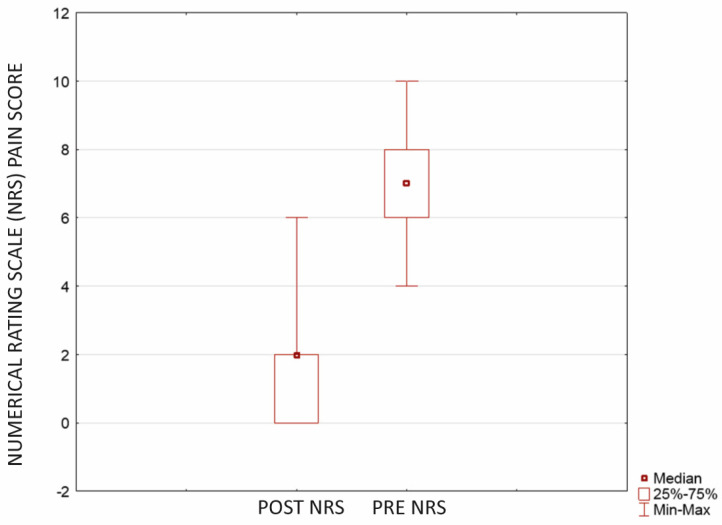
Pain reduction showed with Numerical Rating Scale (NRS) score after microfractures and cartilage reconstruction using a hyaluronate−based membrane as a scaffold (*p* < 0.0001). The mean evaluation time was 23.9 months after surgery (min-max 18.00−27.00 months, SD 1.84).

Our study revealed that the described procedure significantly reduces pain and improves patients’ functions. The NRS score showed a statistically significant pain reduction with Wilcoxone. The preoperative mean NRS score was 7.21 (±0.74; CI = 95%; SD 1.93, median 7.00; range 4.00–10.00) versus 1.83 (±0.76; CI = 95%; SD 1.98, median 2.00; range 0.00–6.00) postoperatively (*p* < 0.0001) (Figure 1).

**Figure 2:**
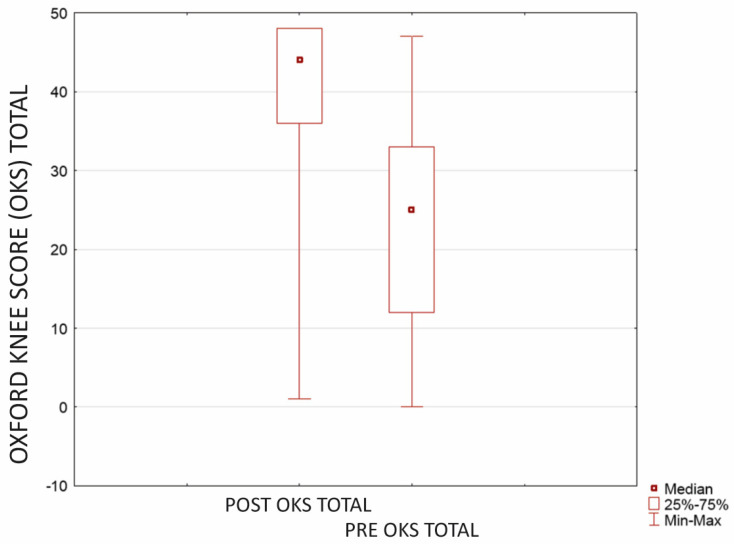
Improvement in patients’ functioning with OKS questionnaire after microfractures and cartilage reconstruction using a hyaluronate−based membrane as a scaffold (*p* < 0.0001). The mean evaluation time was 23.9 months after surgery (min-max 18.00–27.00 months, SD 1.84).

In addition to a substantial pain reduction, the results confirm a significant improvement in patients’ functioning. Functional self-assessment revealed a statistically significant improvement, evident in both (*p* < 0.0001 in both). Before the surgery, the mean score in the OKS questionnaire was 23.1 (±4.59; CI = 95%; SD 12.30, median 25.00; range 0.00–47.00). Postoperatively, OKS improved to 40.2 (±4.02; CI = 95%; SD 10.76, median 44.00; range 1.00–48.00) (Figure 2).

**Figure 3:**
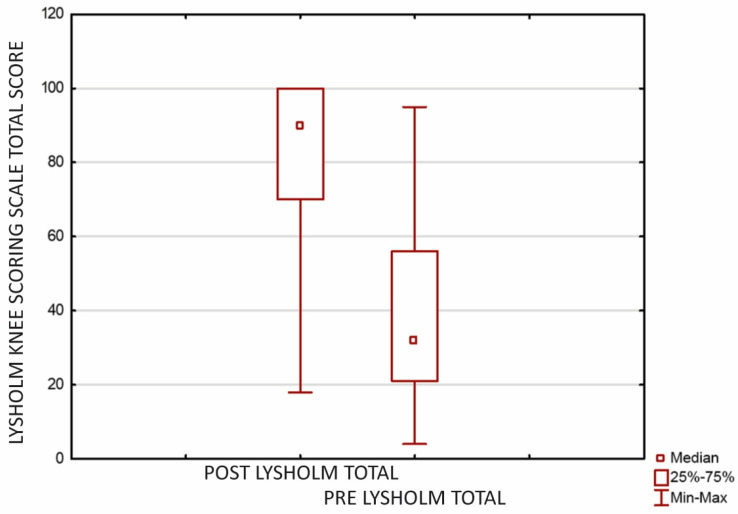
Improvement in patients’ functioning assessed using the Lysholm Knee Scoring Scale after microfractures and cartilage reconstruction using a hyaluronate−based membrane as a scaffold (*p* < 0.0001). The mean evaluation time was 23.9 months after surgery (min-max 18.00–27.00 months, SD 1.84).

Statistically significant functional improvement was confirmed using the Lysholm Knee Scoring Scale as well. Before the surgery, the mean total score was 40.38 (±9.67; CI = 95%; SD 25.43, median 32.00; range 4.00–95.00), when postoperatively it improved to 82.38 (±8.21; CI = 95%; SD 21.59, median 90.00; range 18.00–100.00) (Figure 3).

A researcher-made original questionnaire assessed postoperative voluntary vitamin D supplementation. Although not specifically recommended by the clinician, most of the study group did supplement with vitamin D (Table 1).

However, no influence of supplementation in the U Mann–Whitney test was shown, either on postoperative pain (*p* = 0.474) or function (postoperative Lysholm overall score, *p* = 0.573; postoperative OKS, *p* = 0.816).

The course of postoperative rehabilitation was self-assessed by participants of the study group (Table 2).

Similarly, no statistically significant differences in postoperative pain reduction (*p* = 0.474) or function (correlation with postoperative Lysholm overall score *p* = 0.573) in the U Mann–Whitney test were found.

Moreover, this study shows no gender-dependent differences, neither in preoperative nor in postoperative pain and functional scores. Pain severity, expressed by the NRS, was not gender-related, either before surgery (*p* = 0.349) or after surgery (*p* = 0.584). Similarly, no statistically significant differences in OKS and Lysholm scores between gender were observed either before operation (*p* = 0.074; *p* = 0.357, respectively) or postoperatively (*p* = 0.614; *p* = 0726, respectively).

## 3. Discussion

Injuries in the knee’s internal structures are considered one of the major causes of OA. Patients experiencing knee joint discomfort require proper diagnostics since damaged structures should be treated [7]. However, many intra-articular injuries do not cause substantial complaints that would lead a patient to initiate the diagnostic process. A study published by Horga et al. evaluated 3T MRIs of both knees in 115 subjects who reported no complaints or history of injury. Abnormalities were found in as many as 97% of joints, with articular cartilage damage being the most common (57% of the knees) [8].

Authors of numerous publications, summarized in a systematic review on intra-articular injuries in asymptomatic patients, came to similar findings [9,10,11]. Various pathologies and signs of early OA were commonly found among asymptomatic patients. Nearly a quarter of assessed knees had at least one cartilage defect. The prevalence of chondral lesions was found to increase with age and to be more frequent in females [12].

Cartilage lesions are frequently found as concomitant comorbidities in the diagnostics after an injury, for example, in joint sprains. A retrospective study on a representative (*n* > 25,000) group of patients that underwent diagnostic and therapeutic arthroscopy of the knee joint (due to an acute knee injury, malfunction, or pain and other conditions) showed the presence of cartilage lesions in 60% of cases. A majority of them (65%) were low grade lesions (Outerbridge grade I and II) that were not accounted for as a main reason for complaints [13].

The risk of cartilage damage increases with the stresses on the joint, hence, professional athletes are more prone to this type of injury. Studies evaluating athletes’ joints (40% of which were NBA or NFL players) showed a significantly higher incidence of full-thickness damage compared to the general population. Interestingly, MRIs of half of the asymptomatic athletes showed full thickness chondral damage [14].

An Australian cohort study of patients with symptomatic OA proved that untreated cartilage lesions tend to progress and are associated with worsening of ailment severity over a 2-year period [15]. Moreover, a 4-year follow-up of the study group showed a 6-fold higher risk of requiring total knee arthroplasty among participants with more extensive cartilage damage [16]. Conclusions from the review of the literature by Houck et al. confirm the position that unrepaired cartilage lesions will enlarge, which is already evident at two-year follow-up. However, observations over such a period of time did not demonstrate the occurrence of radiologically overt arthritis [17].

Results obtained in this original study illustrate and prove that damage to the articular cartilage of the knee is a significant and disabling problem. Patients suffer from severe pain, their functioning is markedly limited, they are unable to participate in sport activities, and the frequent activities of daily living are significantly impaired. This research demonstrates that cartilage reconstruction with hyaluronan scaffolding is an effective therapeutic method. It is a one-stage procedure, which reduces the risk of complications and the inconvenience of multi-stage surgeries. In the vast majority of cases, it is performed arthroscopically, without the need for arthrotomy. Reduced tissue traumatization enables a faster and more effective recovery.

The use of a standardized pain assessment tool enabled us to grade patients’ pain. Subgrouping, dependent on the results of pain intensity scales, has been functioning for a long time in the treatment of oncologic patients. It is established that on an 11-point scale (0–10), scores of 0–4 correspond to mild pain, 5–6 to moderate pain, and 7–10 to severe pain [18]. The literature provides numerous adaptations of the subgrouping system for non-oncologic patients. Some of these, summarized in the literature review, set cut-off points between mild and moderate pain at 4, and between moderate and severe pain at 6 [19].

The difference in pre/postoperative pain, achieved with the described method, means a two-grade severity reduction (from severe pain to mild pain). A significant reduction in the severity of pain enables the use of drugs from the lower levels of the analgesic ladder. This means that patients can reduce or completely abandon opioid drugs. Aside from improving quality of life, minimizing the use of pain medications will significantly reduce the risk of side effects. By reducing the symptoms, appropriately selected physiotherapy may be sufficient as pain relief treatment.

Postoperative rehabilitation is an important factor in improving and refining the function of the musculoskeletal system. Interestingly, despite significant differences in either duration, frequency, and form of rehabilitation, no statistical significance was found. The improvement in function, as reported by patients, was significant. The data obtained do not diminish the relevance of postoperative rehabilitation, but prompt further research. Postoperative rehabilitation is a critical component of the therapeutic process; hence, it should be carried out according to a well thought out plan. This is particularly important for patients with high demands, such as professional athletes. The aim of treatment is to restore joint function and decelerate the progression of osteoarthritis. The pace of rehabilitation varies depending on the patient and his general and local condition. Hence, the rehabilitation protocol should not be standardized, but rather drawn up individually [20]. This study is limited, with no possibility to compare individuals that underwent a ‘standardized’ rehabilitation against others who did not, which may bias the results. This demands further research with patients grouped according to different rehabilitation protocols.

Due to the existing literature reports indicating an association between vitamin D and articular cartilage, we retrospectively evaluated the effect of postoperative supplementation on outcomes. We evaluated spontaneous postoperative supplementation that was not recommended by the supervising physician.

Studies on rats have shown that vitamin D supplementation has a positive effect on articular cartilage thickness, glycosaminoglycan levels, and collagen fiber density, while a vitamin D-deficient diet has a detrimental effect on knee cartilage composition, causing a significant decrease in proteoglycan content [21,22]. Vitamin D deficiency is linked to elevated proteolytic enzyme activity, while supplementation reduces metalloproteinase activity and exhibits chondroprotective effects [23]. These findings are relevant for populations in latitudes where sunlight is insufficient for endogenous vitamin D synthesis, leading to widespread deficiency [24,25]. Human studies confirm the association between vitamin D levels and cartilage thickness. An ultrasound assessment in females reveals a significant reduction in cartilage thickness with severe vitamin D deficiency (plasma vitamin D levels <10 ng/mL) [26]. Long-term vitamin D supplementation slows osteoarthritis progression, as observed in a cohort study using MRI, with effects dependent on supplementation frequency and doses [27].

In our assessment we found no statistically significant association between vitamin D supplementation and clinical improvement, as the effects were equally favorable in both subgroups of patients.

Nonetheless, these results must be interpreted with caution and a number of limitations should be considered in the light of some limitations. First, the study group was not numerous. Despite proving strong relationships, a larger sample would reduce the risk of bias. The second limitation is the fact that, as it was a retrospective assessment, all of the obtained data were self-report. Data may be biased due to the non-remembrance of study participants. The third limitation is the varying time from surgery to data collection (patients were operated on between 2017 and 2022). The average evaluation time for the group was 23.9 months, varying for the patients operated on earliest and latest—7 months (3 patients) and 18 months (2 patients), respectively. All the other patients were evaluated 24 months after surgery. The mean evaluation time for the whole study group was 23.9 months after surgery (min-max 18.00–27.00 months, SD 1.84).

This research is also limited by the lack of data on vitamin D serum levels, hence, it cannot be determined whether it was sufficient or not, both pre- and postoperatively. Additionally, patients supplemented various vitamin D products, such that variability could bias the results. Taking the aforementioned limitations into account, it is necessary to conduct further research with a control and a study group that supplement defined doses of vitamin D homogeneously regarding health conditions affecting its absorption. It may be possible that a prospective study will result in different findings.

The results obtained in this study clearly support the effectiveness of osteochondral reconstruction using hyaluronate-based scaffolds. The achieved functional improvement and substantial pain reduction are crucial for patients’ well-being. From a long-term point of view, the treatment of cartilage injuries significantly delays development of osteoarthritis. Considering the single-stage and minimally invasive nature of this procedure, the authors suggest it to be more widely used.

## 4. Materials and Methods

In a qualitative descriptive study, we retrospectively assessed the functional outcome and pain reduction among the patients that underwent arthroscopic surgery due to osteochondral lesion of the knee. All of them were operated on in the Clinic of Orthopedics and Traumatology, University Clinical Hospital in Wroclaw, between 2017–2022.

A total of 32 patients were eligible to take part in the study; finally, 29 of them consented and were included. One person was excluded because they underwent two surgeries on the same knee and the other two refused to participate. Finally, the study group counted 13 females and 16 males. At the time of the surgery study, the group age was, on average, 40.9677 years old (min-max 18–65 years, SD 14.39556) and had a BMI of 26.8227 kg/m^2^ (min-max 19.90–36.48, SD 7476). The average time of surgery was 110.16 min (min-max 55.00–175.00 min, SD 33.39). Patients stayed at the hospital for minimum 1 and maximum 5 days (mean 2.45 days, SD 1.26).

Evaluation of the patients was performed between 2020 and 2023. Patients were evaluated after a time of at least 18 months, while the patients operated earliest were evaluated at 27 months after surgery and those operated latest were assessed at 18 months after surgery. All the other patients were evaluated 24 months after surgery. The mean evaluation time was 23.9 months after surgery (min-max 18.00–27.00 months, SD 1.84).

### 4.1. Procedure

The procedure consisted of lesion debridement, microfractures, and cartilage reconstruction using a hyaluronate-based membrane as a scaffold (HYALOFAST^®^, Anika Therapeutics, Inc., Bedford, MS, USA) fixed with a tissue glue. All of the surgeries were performed arthroscopically and none of them required arthrotomy. Each surgery was performed by one of three orthopedic surgeons highly experienced in knee arthroscopy.

**Figure 4:**
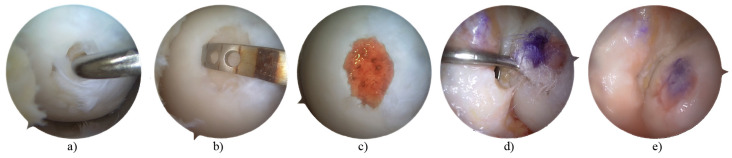
Described procedure. Cartilage lesion inspection (**a**); debridement and measurement (**b**); microfractures (**c**); implantation of scaffold–dry arthroscopy (**d**); scaffold augmentation with tissue glue (**e**).

The first part of the surgery (joint inspection, debridement, microfractures) was performed using the wet-arthroscopy technique, whereas implantation and fixation of the scaffold were performed using the dry-arthroscopy technique (Figure 4).

### 4.2. Assessments

The study group self-assessment was based on widely used questionnaires: the Numerous Rating Scale (NRS), Oxford Knee Score (OKS), and Lysholm Knee Scoring Scale. In addition to these scales, all the participants fulfilled two questionnaires compiled specifically for this study. Those questionnaires were based on self-assessment on postoperative rehabilitation and voluntary postoperative vitamin D intake. The questionnaires were completed by the researcher during teleconsultation or a face-to-face post-operative follow-up.

Patients were asked to fulfill the NRS, OKS, and Lysholm Knee Scoring Scales twice, regarding their condition before surgery and currently.

All the medical data from the day the patients were admitted to the hospital were obtained.

### 4.3. Analysis

Statistical analyses were performed using TIBCO Software Inc. (2017). Statistica (data analysis software system), version 13.3 PL. All analyses were performed on the total population. Descriptive analyses were used to describe the baseline characteristics. All data were analyzed for Gaussian distribution using the Shapiro–Wilk and Kolmogorov–Smirnov normality tests. The U Mann–Whitney test for two independent samples was used because values were not normally distributed. To calculate the difference between sets of pairs and analyze these differences to establish if they are statistically significantly different from one another, the Wilcoxon test was used. For correlation analyses, the Spearman’s Rho correlation test was used. In all calculations the statistical significance was considered at *p* < 0.05.

## Figures and Tables

**Table 1:** Voluntary vitamin D supplementation during postoperative period.

Variable 1	Value 1	No. (%)	Value 2	No. (%)	Value 3	No. (%)	Value 4	No. (%)	Value 5	No. (%)
Vitamin D supplementation	Yes	19 (65.52%)	No	10 (34.48%)						
Supplemented dose	0–1000 u/d	1 (3.45%)	1001–2000 u/d	6 (20.69%)	2001–4000 u/d	8 (27.59%)	4001–10,000 u/d	3 (10.34%)	>10,000 u/d	1 (3.45%)
Supplementation period after surgery	1 (3.45%)	2 (6.90%)	1–3 months	5 (17.24%)	3–6 months	5 (17.24%)	6–12 months	0 (0.00%)	>12 months	7 (24.14%)
Relation with a meal	on an empty stomach	4 (13.79%)	before a meal but not on an empty stomach	0 (0.00%)	during a meal	2 (6.90%)	up to 30 min after a meal	7 (24.14%)	independent from a meal	6 (20.69%)
Elimination diet	vegetarian	4 (13.79%)	vegan	0 (0.00%)	gluten-free	2 (6.90%)	lactose-free	0 (0.00%)	other	0 (0.00%)

**Table 2:** Rehabilitation during postoperative period.

Variable 1	Value 1	No. (%)	Value 2	No. (%)	Value 3	No. (%)	Value 4	No. (%)	Value 5	No. (%)
Rehabilitation up to 12 months after surgery	Yes	29 (100%)	No	0 (00.00%)						
Gap between surgery and first rehabilitation	<2 weeks	7 (24.14%)	2–4 weeks	10 (34.48%)	4–12 weeks	10 (34.48%)	12–24 weeks	1 (3.45%)	>24 weeks	1 (3.45%)
Duration of rehabilitation	<1 month	5 (17.24%)	1–3 months	9 (31.03%)	3–6 months	8 (27.59%)	6–12 months	3 (10.34%)	>12 months	4 (13.79%)
Frequency of sessions (per week)	less that once	0 (00.00%)	once	7 (24.14%)	2 times	8 (27.59%)	3 times	11 (37.93%)	>3 times	3 (10.34%)
Types of applied treatments	physical therapy	17 (58.62%)	kinesiotherapy	12 (41.38%)	CPM	3 (10.34%)	manual therapy	17 (58.62%)	other	2 (6.90%)

## Data Availability

The data presented in this study are available on request from the corresponding author. The data are not publicly available.
